# Sociodemographic determinants of glycaemic control among children with type 1 diabetes in South Eastern Nigeria

**DOI:** 10.11604/pamj.2021.38.250.19790

**Published:** 2021-03-09

**Authors:** Chinwe Flora Ogugua, Ugo Nnenna Chikani, Chikosolu Yvonne Okiche, Ugochi Maryann Ibekwe

**Affiliations:** 1 Paediatric Endocrinology Unit, Department of Paediatrics, Alex Ekwueme Federal University Teaching Hospital Abakaliki, Abakaliki, Nigeria,; 2 Paediatric Endocrinology Unit, Department of Paediatrics, University of Nigeria, Ituku Ozalla Campus, Enugu, Nigeria,; 3 Department of Paediatrics, Ebonyi State University, Abakaliki, Ebonyi, Nigeria

**Keywords:** Diabetes, glycaemic control, sociodemographic factors

## Abstract

**Introduction:**

diabetic complications have been identified as the major causes of morbidity and mortality in persons with type 1 diabetes mellitus (T1DM). Lack of appropriate glycaemic control is a significant risk factor for the onset and progression of long term complications of diabetes. Identifying the determinants of good glycaemic control is therefore imperative.

**Methods:**

this was a cross-sectional, hospital-based study of children aged 3-18 years with T1DM. Subjects were consecutively enrolled after obtaining consent from their parents and assent from children aged ≥7 years. A questionnaire was completed recording their clinical history and sociodemographic variables. Their HbA1c was estimated and values ≤7.5% was defined as the cut-off for optimal glycaemic control.

**Results:**

seventy-one children with T1DM were enrolled for the study. Thirty-eight (53.5%) of them were males. Mean age (years) was 13.7±4. Mean age at onset of diabetes was 11.6 years (range: 3-16 years), mean duration of diabetes was 24.4 months (range: 4-84 months), mean HbA1c value was 10.5% (range: 6.4%-14%); a multivariate logistic regression analysis was performed to identify determinants of optimal glycaemic control. Only caregivers' involvement in diabetes management P<0.016, odd ratio 13.03 (95% CI: 1.60-105.95) was identified as determinant of good glycaemic control.

**Conclusion:**

our data suggest that of all the sociodemographic factors studied, caregivers' involvement in diabetes management was the only strong determinant for optimal glycaemic control.

## Introduction

The burden of type 1 diabetes mellitus is increasing in the sub-Saharan Africa in the face of poor health care financing. Out-of-pocket payment for health services by patients greatly limit their ability to purchase insulin and test strips for their diabetes management. This results in poor glycaemic control and increased mortality in children living with type 1 diabetes mellitus (T1DM). Diabetes related complications have been identified as the major cause of morbidity and mortality in persons with T1DM. Optimal glycaemic control has consistently been associated with fewer and delayed microvascular complications. Monitoring and achieving optimal glycaemic control is a critical therapeutic goal in the management of diabetes mellitus [1]. Lack of appropriate glycaemic control is a significant risk factor for the onset and progression of complications of diabetes mellitus. The epidemiology of diabetes interventions and complications (EDIC) study and other related studies, showed that 5-7 years of poor glycaemic control, even during adolescence and young adulthood, results in an increased risk for microvascular and macrovascular complications in the subsequent 6-10 years [2-6]. This supports the need to maintain optimal glycaemic control. There are several measures of optimal glycaemic control; these include measurement of immediate glycaemic control as best determined by self-monitoring of blood glucose (SMBG). Self-monitoring of blood glucose provides immediate documentation of hyperglycaemia and hypoglycaemia, which allows implementation of strategies to optimally treat, as well as to avoid out of range glucose values [7]. Studies of brain imaging have demonstrated that both hypoglycaemia and hyperglycaemia could cause alterations in the central nervous system (CNS) and these effects could be both acute and chronic [8]. Frequent self-monitoring of blood glucose has been associated with improved HbA1c in patients with type 1 diabetes [6,9]. Most national bodies have recommended good glycaemic control as fasting blood sugar (FBS) level from 70 to 130 mg/dl [1].

Glycated haemoglobin (HbA1c or A1C) estimation is another measure of glycaemic control. HbA1c estimation is the only measure of chronic glycaemic control for which robust outcome data are available [10]. It is a standard index of glycaemic control over the preceding period of 8-12 weeks [11]. The target HbA1c for all age-groups is recommended to be less than 7.5% (58 mmol/mol) [9]. Elevated HbA1c predicts long term microvascular and macrovascular outcomes [4,12]. Furthermore, poor glycaemic control is associated with increased costs of medical care for patients with diabetes [13].

Maintaining optimal glycaemic control is not easily achieved by many patients with type 1 diabetes mellitus. Achieving optimal glycaemic control could be mitigated by several factors which are complex and multi-factorial. However, identifying precisely what factors lead to poor glycaemic control is very challenging as these vary from centre to centre. Studies involving patients with T1DM from different countries have found correlations between poor glycaemic control and factors which include sociodemographic characteristics; diabetes management (insulin therapy, dietary, exercise and blood glucose monitoring), patient related factor like self-care and the interaction between patient and health care providers [1,13,14].

A population-based study by DIABAUD2 a Scottish study group for the care of the young diabetic showed that young age, use of multiple dose insulin regimens, having a sibling with T1DM and children with both parents at home were strong determinants of good glycaemic control [15]. Evidence from the diabetes control and complications trial (DCCT), suggests that poor glycaemic control early in the course of the disease may exert long-lasting effects on the risk for diabetes-related complications (i.e. the metabolic memory hypothesis) [16]. Non-Hispanic black children with type 1 diabetes experience significantly greater deterioration over time than non-Hispanic white children [17]. Therefore, identifying determinants of good glycaemic control is important in order to proactively tailor interventions toward them, hence prevent deterioration in glycaemic control and eventual development of microvascular complications.

Alex Ekwueme Federal University Teaching Hospital Abakaliki runs a free diabetes care policy (free insulin, glucose monitoring test kits and HbA1c evaluation) for all children and young adults with T1DM, thus providing an environment that should facilitate equal health outcomes in patients independent of background, sex or socioeconomic status. Despite the free diabetes care and intensive diabetes education, most children with T1DM in this centre still have poor glycaemic control and early signs of microvascular complication with short duration of diabetes [18]. Hence, the compelling need to identify other determinants of glycaemic control. Poor glycaemic control has also been observed even in countries that offer health insurance to members, indicating that other factors apart from insulin therapy, play a vital role in achieving optimal glycaemic control [15]. This study was carried out with the aim of identifying the determinants of glycaemic control in children aged 3-18 years with T1DM.

## Methods

This was a cross-sectional hospital-based study carried out at the Alex Ekwueme Federal University Teaching Hospital Abakaliki (AE-FUTHA). AE-FUTHA is the only tertiary hospital in Ebonyi State, as such, provides services to the state and towns in the neighboring 4 states. The hospital practices a free diabetes care policy (free insulin, glucose monitoring test kits and HbA1c evaluation) for children and young adults with T1DM. Patients seen in the paediatric endocrinology clinic of AE-FUTHA are evaluated by a multidisciplinary team of paediatric residents, paediatric endocrinologist, dietitians, clinical psychologist, social welfare workers and diabetes nurse educators during their first visit as a baseline. During subsequent visits, consultation will depend on needs after evaluation by the paediatric endocrinologist.

**Study participants:** children with type 1 diabetes mellitus (T1DM) aged 3-18 years were consecutively enrolled from the paediatric endocrinology clinic of AE-FUTHA after obtaining parental consent and assent from children aged ≥7 years. Patients on regular follow up for at least 4 months and those who had at least one documented HbA1c level were included in the study while those who were not willing to participate and did not sign the written informed consent were excluded.

### Measures and procedure

**Sociodemographic data:** a questionnaire was completed recording their sociodemographic information. These include: age, sex, socioeconomic status, relationship with caregiver and involvement of care givers in diabetes management. The socioeconomic status was determined using the Oyedeji´s classification [19], which is based on parents/caregivers´ education and occupation. Relationship with caregiver was assessed based on who the child is currently living with (1-if living with parents, 2-mother alone, 3-father alone, 4-extended relations/others). Caregiver´s involvement in diabetes care was assessed based on the number of times they participated in blood glucose testing, injection of insulin and possible insulin adjustment in a week: a) no participation; b) 1-2 times a week; c) 3-4 times a week; d) 5-7 times a week. These represent none involvement, minimal, moderate and active involvement in diabetes management respectively.

**Clinical and anthropometric measures:** diabetes-related information including: age at onset of diabetes; duration of diabetes mellitus and insulin regimen were obtained. Clinical history was obtained and physical examination performed. Blood was taken for HbA1c and fasting blood glucose. Weight was measured without shoes or heavy clothing with a SECCA® scale. It was recorded to the nearest 0.1 kilogram [20]. Height was determined to the nearest 0.1 cm with a SECCA 850 stadiometer following the protocol for height measurement [20]. The weight and height values obtained were used to calculate the body mass index (BMI). The BMI z-score was calculated by comparing each participant´s BMI measure with age and sex-specific standards published by the national centre for health statistics. These standards enabled each participant´s deviation from the reference value to be calculated in terms of a normalized standard deviation score (SDS or z-score). The participants were classified based on the WHO classification as underweight (SDS<-2), normal weight (SDS>-2 to<+2) and overweight (SDS>+2).

Glycosylated haemoglobin (HbA1c) was estimated and the mean HbA1c over a year period was calculated. The HbA1c was measured immunochemically in the clinic using directional coronary atherectomy (DCA) 2000® + (Bayer corporation) [21]. The instrument was standardized against the diabetes control and complication trial method. Quality control using standard solutions was assured.

**Statistical analysis:** information obtained were transferred to electronic data base prepared using Microsoft Office Excel 2007 and statistical analyses performed using the Statistical Package for Social Sciences (SPSS) software for Windows, version 20. Comparison of sociodemographic variables with the glycemic control (dichotomized as poor and good control) was done using Fisher´s exact test and Chi-square test as appropriate. The strength of relationships of variables that were significant at the bivariate level of analysis were further tested using multivariate logistic regression analysis. All test of significance were 2 tailed at 95% confidence interval. P value was considered significant if it is less than 0.05.

**Ethical clearance and consent:** ethical approval was obtained from the Health Research and Ethics Review Committee of the various hospitals with reference number: REC APP NO 25/10/2013-19/02/2014 before commencement of the study. Ethical principle was according to Helsinki declaration. Parental consent and assent from children aged ≥7 years were obtained. Data obtained were encrypted and stored in a file that was only assessable to the researchers to ensure confidentiality of information. Subjects were interviewed individually in a quite office to ensure confidentiality. Participants with poor glycemic level were counselled appropriately.

## Results

**Sociodemographic characteristics:** a total of seventy-one children with T1DM (38 males and 33 females) were included in the study. Mean age was 13.7 ± 4.1 SD. The majority of the participants (67.6%) were between the ages of 13-18 years. Fifty-one (71.8%) of the participants came from low socioeconomic class according to the Oyedeji classification [20]. Most of the children (66.2%) lived with both parents and 60.6% of the care givers were actively involved in the diabetes management of the participants. A large proportion of the participants (85.9%) were not involved in physical exercise. Their activities were limited to routine self-care. The sociodemographic characteristics is shown in Table 1.

**Table 1:** characteristics of participants (N=71)

**Sex**	
Male	38 (53.5%)
Female	33 (46.5%)
**Mean age (years)**	13.7 ± 0.5
<5 yrs	2 (2.8%)
5-12 yrs	21 (29.6%)
13-18 yrs	48 (67.6%)
**BMI z score**	
>+2 SD	1 (1.4%)
-2 to +2 SD	47 (66.2%)
<-2 SD	23 (32.4%)
**Socioeconomic status**	
Low SEC	51 (71.8%)
Upper SEC	20 (28.2%)
**Relationship with caregiver**	
Both parents	47 (66.2%)
Single parent	18 (25.4%)
Relatives	16 (22.5%)
**Involvement of caregivers in DM management**	
Active involvement	43 (60.6%)
No active involvement	28 (39.4%)
**Exercise programmers**	
Yes	10 (14.1%)
No	61 (85.9%)
**Types of insulin**	
Premixed insulin	30/70 59 (83.1%)
Intermediate/rapid acting insulin	7 (9.9%)
Long/fast acting insulin	5 (7%)
Mean age at onset of diabetes	12.6 yrs (range: 2 - 16 yrs)
Mean duration of diabetes	24.4months (range: 3 - 84 months)
Mean HbA1c	10.5% (range 6.4 - 14%)
≤ 7.5% (<58mmol/mol)	15 (21.1%)
>7.5%	56 (78.9%)

**Glycaemic control:** the cut-off for optimal glycaemic control was set at ≤7.5% (≤58mmol/mol). Of the 71 participants, 15 (21.1%) had optimal glycaemic control while a significant proportion of the children; 56 (78.9%) where poorly controlled. The mean HbA1c was 10.5% (range 6.4 - 14%). The mean age at onset of diabetes was 12.6 ± 4 SD (range 2 - 16 years) and the mean duration of diabetes mellitus was 24.4 months (range 3 - 84 months). Most of the participants 59 (83.1%) were receiving premixed insulin (30/70) which is the free insulin provided by the hospital management, while the rest used intermediate with rapid acting insulin or analogs (Table 1).

Comparison of sociodemographic variables with the glycemic control (dichotomized as poor (HbA1c >7.5) and good control (HbA1c <7.5) was done using Fisher´s exact test and Chi-square test as appropriate. The analysis demonstrated that patients´ age, care givers´ involvement in diabetes management (p<0.003 LR=10.3), age at onset of diabetes (p<0.04 LR=6.1) and duration of diabetes (p<0.04 LR=4.8) respectively were strong determinants of glycaemic control (P<0.0001 LR=13.8). Gender, primary care givers, insulin formulation, exercise, BMI and SEC, showed no statistically significant differences (Table 2). The strength of relationships of variables that were significant at the bivariate level of analysis were further tested using multivariate logistic regression analysis. Only caregivers´ involvement in diabetes management was significant. P<0.016, odd ratio 13.03(95% CI: 1.60-105.95) (Table 3).

**Table 2:** relationship between HbA1c and sociodemographic/clinical variables

Covariates	Number of children	Number of children	p-value
	With HbA1C<7.5%	With HbA1C>7.5%	
**Sex**			
Male	11 (28.9%)	27 (71.1%)	
Female	4 (12.1%)	29 (87.9%)	0.073*
**Age (years)**			
<5 years	1 (50%)	1 (50%)	
5-12	10 (47.6%)	11 (52.4%)	
13-19	(48.3%)	44 (91.7%)	0.001*
**Duration of diabetes**			
<2 years	14 (26.9%)	38 (73.1%)	
>2 years	1 (5.3%)	13 (94.7%)	0.04*
**Age at onset of TIDM**			
<5 years	1 (20%)	4 (80%)	
5-12 years	11 (33.3%)	22 (66.7%)	
13-19 years	3 (9.1%)	30 (94.9%)	0.04*
**Socioeconomic class**			
Lower Sec	9 (27.9%)	42 (71.1%)	
Upper Sec	6 (20.0%)	14 (80%)	0.20**
**BMI z score**			
>+2 SD	1 (50%)	1 (50%)	
-2 to +2 SD	13 (29.5%)	32 (71.7%)	
<-2 SD	1 (10%)	22 (90%)	0.30*
**Relationship with caregiver**			
Both parents	10 (27%)	27 (73%)	
A parent	4 (22.2%)	14 (77.8%)	
A relation	1 (6.3%)	15 (93.7%)	0.003*
**Caregiver’s involvement in**			
**Diabetes management**			
Active involvement	14 (32.6%)	29 (67.4%)	
No active involvement	1 (3.6%)	27 (96.4%)	0.003*
**Involvement in exercise**			
Regular exercise	3 (3%)	7 (70%)	
No exercise	12 (10.7%)	49 (80.30%)	0.04*
**Type of insulin used by**			
**Patients**			
Premixed 30/70	14 (23.7%)	45 (76.3%)	
Intermediate and short acting	0 (0.0%)	7 (100%)	
Long and rapid acting	1 (20%)	4 (80%)	0.37*

Fisher exact test*; Chi square**

**Table 3:** multiple logistic regression of the significant variables

Variables	Odd ratio (OR)	95% confidence interval	p-value
**Age**			
<5 years	reference		
5 - 12	1.10	0.06 - 20.01	0.95
13 - 19	11.00	0.57 - 211.17	0.11
**Duration of illness**			
>2 years	reference		
<2 years	6.63	0.81 - 54.42	0.07
**Age at onset (years)**			
<5	reference		
5-12	0.50	0.05 - 5.02	0.56
13-19	0.25	0.03 - 2.40	0.23
**Caregivers' involvement**			
Active involvement	reference		
No active involvement	13.03	1.60 - 105.95	0.016

NB: Dependent variable (0=good/1=poor glycemic control)

## Discussion

Achieving optimal glycaemic control is a critical therapeutic goal in the management of diabetes mellitus [1]. In the present study, the overall glycaemic control was poor with a mean HbA1c of 10.5% which is similar to values reported in other African studies [21-26]. Twenty-one percent (21.1%) of the participants achieved good glycaemic control (HbA1c <7.5% i.e. <58mmol/mol) while most of the participants (78.9%) had poor glycaemic control (HbA1c >7.5%) using the international society for pediatric and adolescent diabetes (ISPAD) recommendations [9]. The percentage of participants with good glycaemic control was also comparable to the findings of Ngwiri *et al*. [24] in Kenya and Gebre-Yohannes and Rahlenbeck in Ethiopia [25]. This poor glycaemic control was seen despite regular supply of insulin, suggesting that factors other than availability of insulin could play a part in glycaemic control.

In this study, four important factors: young age, duration of diabetes less than 2 years, young age at onset of diabetes and care givers involvement in diabetes were identified as determinants of optimal glycaemic control at the bivariate level of analysis. The strength of their relationship was further subjected to multiple logistic regression, caregivers´ involvement in the care was the only significant predictor of good glycemic control. This is such that participants with no active caregiver involvement in their care were 13 times more likely to have poor glycemic control. Social support is the single most important moderator of disease outcome especially in chronic diseases like diabetes mellitus, mental illnesses, oncology and haematological diseases [26]. Active caregiver´s involvement consists of the frequency in which the caregiver assists, encourages and supervises in the injection of daily insulin, adherence to blood sugar monitoring, dietary modification and exercise.

It is a secondary modulator of health outcome, whose primary effect involves compliance to medications with resultant positive health outcome. This is because diabetes management is patient-centered, it is beyond availability of insulin and monitoring of glucose, rather it consists of adherence to a demanding regimen that includes multiple insulin injections (or pump therapy) and monitoring blood glucose concentrations four or more times daily required for good glycemic control. This can be a herculin task especially when left only for the patients to perform. An active caregiver who gives emotional, psychological and physical support to patients, who directly observes treatment is more likely to achieve a good disease outcome to a large extent and cannot be over emphasized. Studies in other areas of medicine have equally shown that patients living with chronic diseases need the active involvement of caregivers to have a better disease outcome [26]. Baasher *et al*. as well as Shelton *et al*. reported that the majority of vagrant psychotic patients are usually associated with a dysfunctional family structure and complete lack of caregiver support [27,28].

Similarly, a study conducted in Kenya [24] reported better glycaemic control in children whose both parents were actively involved in their diabetes management. Relationship of the caregiver with the child did not have significant impact on the glycaemic control. Instead, dedication rather than mere blood ties was more pertinent in the management of persons with diabetes [24]. Relationship with caregiver showed no statistically significant association with glycaemic control even in our study. Duration of diabetes and age at diagnosis were not statistically significant as determinants of good glycemic control. Although, most of the children (93.3%) with HbA1c <7.5% had diabetes duration less than 2 years. This may in part be explained by the possibility of some of these patients being in the honeymoon period. In addition, recently diagnosed individuals may still be enthusiastic about their diabetes management and are yet to develop emotional, physical and financial burnt-out syndromes. These burnt-out syndromes are commonly seen in caregivers and patients living with diabetes for a longer duration and may impact negatively on their diabetes management leading to poor glycaemic control.

Clements *et al*. [17] reported poor glycaemic control in patients diagnosed at older ages. They noted specifically that despite achieving better initial glycaemic control after the initiation of insulin therapy, these children experienced greater deterioration in glycaemic control during the first 5 years after diagnosis than younger patients and this occurred despite stricter glycaemic control among older children during the study period. The poorer glycaemic control noted in the older age group could be explained partly by the relative insulin resistance imposed by the pubertal hormones and partly by juvenile delinquency. The older children may be rebellious hence not strictly compliant with their medications and SBGM.

In this study, gender, socioeconomic class, body mass index, involvement in exercise and insulin formulation had no statistically significant impact on their glycaemic control. In contrast to previous studies [29-32] that reported poor glycaemic control in females compared to the males. The observed difference in those studies was attributed to a possible effect of the female pubertal hormones. Regular physical exercise is an important component of diabetes management. Although the evidence for a positive effect of exercise upon glycaemic control (i.e. HbA1c) is weak, there is growing evidence of the benefits of regular physical activity upon cardiovascular risk factors [33-35]. Participants in this study were mostly sedentary and impact of exercise on glycaemic control was not statistically significant further supporting the findings of Robertson *et al*. [35].

The DIABAUD2 study [15], observed that the use of multiple dose insulin regimen was associated with better glycaemic control. In this study, 83.1% of the participants were on the premix twice daily insulin regimen while the rest on multiple dose insulin regimen. The disproportionate insulin regimen may in part have accounted for the non-significant statistical outcome. Largely, the socioeconomic class didn´t have significant effect on the glycaemic control; perhaps it could be partly due to the free diabetes care policy practiced in the center. The out-of-pocket expenditure is the greatest burden to many families as most of their income is spent on health care. An average of 50-100 dollars per month is needed for insulin and blood glucose strips depending on the dosages and types of insulin. This amount is equal to or more than the minimum wage of an average Nigerian civil servant thus, making diabetes care unaffordable to the majority of the patients. This burden is completely alleviated by the free diabetes care policy, giving equal access to diabetes care to all the children irrespective of their socioeconomic class.

**Disclosure:** the abstract of this article was published in the abstract proceedings of the Royal College of Paediatrics and Child Health Conference which took place on the 13^th^ to 15^th^ May, 2019 at ICC Brimingham United Kingdom [36].

## Conclusion

Of all the sociodemographic determinants studied, caregivers´ involvement was the single most significant moderator of optimal glycaemic control.

### What is known about this topic

- Different studies in systematic review showed that good glycaemic control is achieved in less than 50% of diabetic patients;
- Some studies have suggested insulin therapy knowledge and skill deficit; poor adherence to insulin regimen, self-care, exercise and dietary plan combined with poor interaction between the patient and health care providers as part of the determinants of poor glycaemic control;
- DIABAUD2 a Scottish study group for the care of the young diabetic showed that young age, use of multiple dose insulin regimens and having a sibling with T1DM were strong determinants of good glycaemic control.

### What this study adds

- Besides the availability of insulin, social support (caregivers’ involvement) is the single most important moderator of outcome of all other factors;
- Active involvement of caregivers in diabetes management was a strong determinant for better glycaemic control. Dedication rather than mere blood ties was more pertinent to diabetes management (this was written because there was no significant relationship between blood relationship and good glycemic. Mere blood relations offered no added advantage to blood sugar control).
